# The Power of Clinical Diagnosis for Deciphering Complex Genetic Mechanisms in Rare Diseases

**DOI:** 10.3390/genes14010196

**Published:** 2023-01-12

**Authors:** Li Shu, Tatiana Maroilley, Maja Tarailo-Graovac

**Affiliations:** 1 Departments of Biochemistry, Molecular Biology and Medical Genetics, Cumming School of Medicine, University of Calgary, Calgary, AB T2N 4N1, Canada; 2 Alberta Children’s Hospital Research Institute, University of Calgary, Calgary, AB T2N 4N1, Canada

**Keywords:** clinical diagnosis, rare disease, complex mechanism, structural variants, non-coding variants

## Abstract

Complex genetic disease mechanisms, such as structural or non-coding variants, currently pose a substantial difficulty in frontline diagnostic tests. They thus may account for most unsolved rare disease patients regardless of the clinical phenotype. However, the clinical diagnosis can narrow the genetic focus to just a couple of genes for patients with well-established syndromes defined by prominent physical and/or unique biochemical phenotypes, allowing deeper analyses to consider complex genetic origin. Then, clinical-diagnosis-driven genome sequencing strategies may expedite the development of testing and analytical methods to account for complex disease mechanisms as well as to advance functional assays for the confirmation of complex variants, clinical management, and the development of new therapies.

## 1. Introduction

Rare diseases (RDs) are defined as “diseases with no more than 5 out of 10,000 people affected”, and 80% of RDs are of genetic origin [1,2,3]. Because of the clinical heterogeneities of RDs, genetic analysis relying on clinical phenotypes is difficult in many instances. The same phenotype may result from different genetic causes, while different phenotype combinations may lead to different gene priorities in genetic diagnosis.

Many new global efforts are focusing on undiagnosed RD patients [4]. Different strategies involving various technologies and approaches are applied to either uncover new gene-disease associations or more complex genetic mechanisms of a disease, such as complex genome rearrangements, variants in non-coding regions, mosaic variants, epigenetic variants or variants affecting more than one gene (e.g., digenic inheritance). However, the strategies for unravelling genetic causes in undiagnosed RD patients continue to be dominated by methods that focus almost exclusively on coding regions, either technological method (exome-based sequencing or arrays) and/or analytical methods (bioinformatics analyses focused on coding regions). Consequently, the majority of the likely pathogenic or pathogenic variants in the ClinVar (access https://www.ncbi.nlm.nih.gov/clinvar/ accessed on 15 November 2022) are single nucleotide variants (SNVs), followed by copy number variants (CNVs) or Indels affecting the protein-coding regions [5]. Importantly, the predominant focus on protein-coding regions may be impacting the discovery of novel gene-disease associations, as the mechanisms of the disease may be more complex and refractory to current discovery methods [6,7,8]. It has been estimated that fewer than 40% of the genetic causes of RDs could be explained by coding variants, meaning a large proportion of complex genetic mechanisms is under-explored [9].

The clinical-diagnosis-driven strategy focuses on well-known disease-associated genes in patients with particular pathologies. Being causative gene oriented, this strategy has uncovered most reported regulatory variants [10]. The clinical diagnosis of a known Mendelian disease combines multiple diagnostic criteria based on prominent phenotypes or biochemical hallmark signs which point toward a specific gene or a genetic pathway. For example, the typical clinical phenotypes of Salla disease (SD; OMIM: #604369) caused by *SLC17A5* mutations are hypotonia, ataxia, nystagmus, epilepsy, and findings of cerebral and cerebellar atrophy accompanied by an elevation of free urinary sialic acid [11]. SD and other similar RDs have a minimal causative gene range; most are monogenic. An appreciable number of clinically diagnosed cases, albeit with no or partial genetic diagnosis, harbor types of variants (e.g., non-coding or structural variants) in known causative genes that are refractory to identification by current clinical molecular diagnostic paradigms [12].

Therefore, strongly suspected clinical diagnosis can give us vital clues to characterize unsuspected disease-causing variants by the application of new genomic and analytic techniques to a narrow range of causative genes. It has a unique advantage in helping dig into more complex genetic mechanisms by accurately pinpointing the causative genes in previously ‘undiagnosed’ or ‘partially diagnosed’ patients. In turn, solving these complex genetic mechanisms of well-defined clinical phenotypes has the potential to advance genome-wide sequencing and analytical strategies for many unsolved RD patients regardless of the clinical phenotype.

## 2. Unravelling Complex Genetic Mechanisms of RDs Based on Clinical Diagnosis

It is not unusual for a patient with a well-defined clinical diagnosis of an autosomal recessive disease to only receive a partial or inconclusive genetic diagnosis (a single detectable heterozygous variant [13]) or for a patient with a clinically well-characterized dominant RD to receive no genetic diagnosis (no detectable variants in the gene known to be associated with the disease) [14,15]. Along the lower-than-anticipated diagnostic yield of genome-wide sequencing approaches, the patients with a well-defined clinical diagnosis but no or partial genetic diagnosis are a clear reminder that we need to find a better way to identify complex genetic mechanisms and increase the diagnostic success by tailored analytical methods.

With technological advances such as short-read genome sequencing (SR-GS), long-read genome sequencing (LRS), optical genome mapping (OGM), transcriptomics (RNA-Seq), and epigenomics analyses, we are equipped with more opportunities to identify unknown causes [16]. However, the main challenge is how to efficiently apply and maybe combine various new methods to make a molecular diagnosis. On the technical level, for sequencing methods such as SR-GS and LRS, new bioinformatic tools are still being developed to identify and interpret variants. The challenges still revolve around identification, interpretability of complex genetic mechanisms, and reproducibility of results [17]. While SR-GS performs well for the identification of SNVs and small indels, it is challenging to adapt to the detection of larger and more complex sequence variants [18]. On the other hand, LRS may help in complex variant detection and especially offers a possibility to detect epigenetic changes; however, current difficulties with data processing speed, elevated need in computational storage, and the cost and the complexity of error correction poses a barrier that needs to be overcome before the LRS is applied in clinical practice [19].

**Figure 1:**
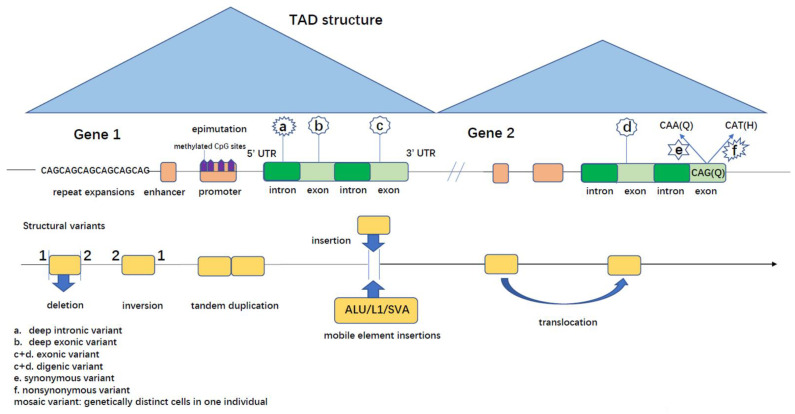
The schematic figure of complex genetic mechanisms.

To assess the power of clinical diagnosis in identifying complex genetic mechanisms, we systematically reviewed the literature covering the spectrum of clinical phenotypes and the genetic methods that led to the successful identification of complex genetic mechanisms. These complex genetic scenarios (Table 1) include SNVs that either affect non-coding regions (e.g., introns, promoters, untranslated regions, regulatory elements) or may be in either coding or non-coding regions, disrupting gene function in a more complex way (e.g., via mobile element insertions, genomic rearrangements, repeat expansions, mosaic variants, oligogenic mechanisms, epigenetic changes). In either case, these variants would not have been accurately captured by routine clinical genetic testing such as exome and Sanger sequencing or clinical arrays (Figure 1). 

### 2.1. Non-Coding Variants in Patients with Established Clinical Phenotypes

#### 2.1.1. Single-Nucleotide Variants

So far, associations of non-coding variants (in less than 0.2 % of individuals) with RD phenotypes have been lagging when compared to coding variants (ClinVar; access https://www.ncbi.nlm.nih.gov/clinvar/ accessed on 15 December 2022). Identifying SNVs in non-coding regions is difficult using routine genetic testing and necessitates the utilization of methods covering the entire genome such as SR-GS [5].

- Deep Intronic Variants

Although various studies have been performed to unravel the intronic variants in clinically diagnosed diseases using extensive sequencing methods, deep intronic variants are still one of the most common missing types. Deep intronic variants lie in the non-coding region of the genome, where some are hundreds or even thousands of base pairs away from the exon-intron junctions (Figure 1). The effects of deep intronic variants on gene expression include pseudo-exon inclusion, competition with canonical splice sites, transcription regulatory motif disruption, as well as non-coding RNA gene inactivation in human diseases [52]. 

The search for missing variants in patients with well-defined clinical diagnoses has been driving the discoveries of deep intronic variants and splicing mechanisms. For example, patients living with the autosomal dominant hereditary hemorrhagic telangiectasia (HHT) (HHT1; OMIM: #187300/HHT2; OMIM: #600376/Juvenile polyposis/HTT syndrome; OMIM: #175050) can remain undiagnosed, despite extensive genetic testing considering the coding regions of known HHT genes. SR-GS identified a deep intronic splicing mutation hotspot in the causative gene *ACVRL1*; seven cases had a deep intronic variant located in intron 9 that disrupted splicing [20] (Table 1). An example of an identification of a deep intronic variant in a partially diagnosed autosomal recessive disease is in the case of ataxia–telangiectasia (AT; OMIM: #208900). After a clinical ataxia gene panel led to a partial molecular diagnosis of a previously reported synonymous pathogenic variant in *ATM*, Maroilley T et al. combined SR-GS and transcript analyses to identify a deep-intronic variant and confirm that it results in the aberrant inclusion of 56 base pairs of the intron in the *ATM* [21] (Table 1).

b. 5′ and 3′ Untranslated Regions (UTRs) Variants

There has been limited research on the variations in the 5′ and 3′ UTRs and their impact on human diseases (Figure 1). Considering their importance in post-transcriptional regulation [53,54], it is not surprising that UTR variants are being identified in genetically unsolved RD cases using the clinical-diagnosis-driven strategy [22,23]. By analyzing targeted sequencing data from neurofibromatosis, type 2 (NF2; OMIM: #101000)—a novel single 5′UTR variant—was identified in the *NF2* gene in two probands, which caused aberrant translational regulation through upstream open reading frames [22] (Table 1). By screening de novo mutations in the 5′ UTR region of *MEF2C* in probands with neurodevelopmental disorder with hypotonia, stereotypic hand movements, and impaired language (NEDHSIL; OMIM: #613443), four variants creating upstream start codons were found [23] (Table 1). The 5′ UTR variant c.-547C > T in the *AR* gene was identified in patients with complete androgen insensitivity syndrome (AIS; OMIM: #300068) using SR-GS after negative results by the Sanger sequencing of the gene coding region [24]. Furthermore, in a proband suspected to have autosomal recessive vasculitis, autoinflammation, immunodeficiency, and hematologic defects syndrome (VAIHS; OMIM: #615688), exome sequencing (ES) only identified one missense variant in the causative gene *ADA2*. Definite molecular diagnosis was made by finding the second variant, a 5′UTR variant in *ADA2,* by further SR-GS analysis [25] (Table 1).

c. Regulatory Elements

Non-coding regulatory elements such as promoters and enhancers can also contribute to a disease through their role in transcription or post-transcriptional regulation mechanisms [55] (Figure 1). The genetic causes of established clinical phenotypes can be helpful in interpreting variants in the regulatory genomic regions. For example, in a patient with the highly specific phenotype aniridia (AN1; OMIM: #106210; typically caused by a haploinsufficiency of *PAX6*) but with negative clinical genetic tests, a de novo SNV was identified in an ultra-conserved cis-element (SIMO enhancer) located 150 kb downstream from *PAX6* [26] (Table 1). The variant was shown to disrupt the *PAX6* binding site and result in the defective expression of *PAX6* [26].

#### 2.1.2. Complex Variants

- Mobile Element Insertions (MEIs)

It has been estimated that over two-thirds of our genome may result from the presence or ancient activity of mobile genetic elements (‘jumping genes’). Mobile element insertions including LINE-1 (or L1), SVA, and Alu are produced through retrotransposition, which mobilize by a copy-and-paste mechanism to different genomic locations and disrupt genetic function [56] (Figure 1). Genome-scale approaches increased the capability to identify new retrotransposon insertions. For example, in a patient with the atypical autosomal recessive sialic acid storage disease (SASDs) [infantile sialic acid storage disease (ISSD; OMIM: #269920)/Salla disease (SD; OMIM: #604369)], a homozygous large (6040 bp) intronic transposal insertion (LINE-1) in intron 9 of *SLC17A5* was identified by ES followed by RNA and genomic DNA analysis after previous negative results by targeted Sanger sequencing [11] (Table 1). The insertion results in aberrant splicing and consequently a frameshift and truncated protein [11]. Similarly, in a patient with a clinical diagnosis of ceroid lipofuscinosis, neuronal, 7 (CLN7; OMIM: #610951), a genetic panel revealed a heterozygous paternally inherited known pathogenic missense variant in *MFSD8*, yet with no second variant identifiable via exome capture. SR-GS analysis revealed maternal inherited 2 kb MEI deep in the intron 6, which was confirmed to be SVA (SINE–VNTR–Alu) retrotransposon [27] (Table 1) that results in the aberrant splicing of the *MFSD8* exon 6 leading to premature translation termination [27].

b. Repeat Expansions (REs)

REs are expansions of sequence repeats with sizes ranging from trinucleotides (CAG, CGG), tetranucleotides (CCTG), pentanucleotides (TGGAA) to even dodecanucleotides [57] (Figure 1). So far, at least 50 RE disorders have been described. However, only half of the expansion loci were recognized in the last ten years, as current widely used molecular diagnostic methods (exome or gene panels) have limited the identification of REs [58]. With the development of bioinformatics tools specifically assessing REs with SR-GS or LRS, it is expected that more RE disorders are to be identified. For example, Kuilenburg et al. identified GCA-REs in the 5′UTR region of the *GLS* by SR-GS. Initial ES only uncovered one heterozygous and damaging variant in *GLS* in two probands who presented with global developmental delay, progressive ataxia, and elevated glutamine (GDPAG; OMIM #618412) [7] (Table 1). Similarly, pathogenic GGC RE in the promoter region of the *XYLT1* gene was identified in eight of ten families with Baratela-Scott syndrome (BSS; OMIM: #615777) with single or no causative variants in *XYLT1* [28] (Table 1). Also, epilepsy, familial adult myoclonic (FAME) is a recently discovered novel RE disorder due to complex RE configurations: a combination of a TTTCA repeat associated with a polymorphic TTTTA repeat [8]. Thus far, six different intronic loci with the pathogenic pentanucleotide RE combination have been described in FAME patients [*SAMD1* [8], *STARD7* [29], *MARCH6* [30], *YEATS2* [31], *TNRC6A* [8] and *RAPGEF2* [8] (Table 1)].

c. Genomic Rearrangements (GRs)

GRs are larger variants (>50 bp) and they regroup into copy number variants (CNVs), such as deletions and duplications; but also structural variants (SVs), such as inversions and translocations; and complex genomic rearrangements (CGRs). While some may be captured by routine clinical diagnostic testing (e.g., arrays), others may be missed (e.g., balanced events such as smaller inversions) [59] (Figure 1). SR-GS has the potential to detect the full spectrum of SVs in a single test [60] with advanced analytical tools, but more knowledge is needed to develop ways to better interpret the impact of GRs on gene function. Multiple examples have shown the success of unravelling SVs in patients with well-characterized clinical diagnoses. For example, in a patient with autosomal recessive dihydropyrimidine dehydrogenase deficiency (DPD deficiency; OMIM: #274270) and single paternally inherited damaging SNV in *DPYD*, SR-GS identified the missing maternal variant, an intragenic inversion (~116 kb) with breakpoints in introns 8 and 12 of the *DPYD* [13] (Table 1). Furthermore, the GRs can affect other non-coding regions in the genome where their impact is more difficult to interpret unless guided by clinical diagnosis and clinical validation. In patients clinically presenting with autosomal recessive Nonaka myopathy (NM; OMIM: #605820), but single heterozygous missense variant only, CNV analyses revealed a deletion in a promoter region of the *GNE* gene. Further gene expression analysis confirmed that the deletion causes reduced GNE expression [32] (Table 1). In a cohort of patients with a clinical diagnosis of autosomal dominant Marshall-Smith syndrome (MRSHSS; OMIM: #602535), a recurrent deletion of the *NFIX* gene was identified using multiplex ligation-dependent probe amplification (MLPA) after negative *NFIX* sequencing [33] (Table 1). After extensive sequencing analysis, three different partial intragenic deletions were identified in *ATP7B* in seven different unexplained autosomal recessive Wilson disease (WD; OMIM: #277900) families using selective amplification and MLPA [34] (Table 1). 

Topologically associating domains (TADs) are megabase-scale genomic regions which are fundamental units of the three-dimensional chromatin structure (Figure 1). The TADs modulate gene expression [10] by limiting the interactions of cis-regulatory sequences to their target genes [61]. Eight different complex SVs have been identified from twenty-two unsolved autosomal-dominant retinitis pigmentosa (RP; OMIM: #268000) families altering gene expression by rearranging TADs and re-wiring enhancer-promoter interactions [35] (Table 1).

d. Epigenetic Changes

There is a growing identification of epivariations in RDs which are most often recognized as promoter hypermethylation events causing gene silencing [62] (Figure 1). Since it is likely to be missed by clinical diagnostic approaches, epigenome profiling or LRS methods that take into consideration DNA modifications could shed light on epivariants. The two main types of epivariants are primary, where in the absence of DNA sequence change stochastic errors in DNA modification affect gene expression [63], and secondary epivariants, which are downstream events that result from an underlying change in the DNA sequence and affect more than one gene—typically referred to as episignature—which could help delimit the genetic causes of some RDs. For instance, in cases with autosomal recessive methylmalonic aciduria and homocystinuria, cobalamin C type (MAHCC; OMIM: #277400), after the identification of a genetic mutation, an epimutation at the *MMACHC* locus consisting of hypermethylated CpG sites, including promoter and first exon, was found secondary to splicing variants in *PRDX1*. The hypermethylation of *MMACHC* was further found to cause the aberrant antisense transcription silencing of *MMACHC* expression [36] (Table 1). 

### 2.2. Coding Variants in Patients with Established Clinical Phenotypes

#### 2.2.1. Single-Nucleotide Variants

Albeit the current methods focused on coding regions are efficiently detecting SNVs in protein coding regions, the variant-interpretation methods may still lead to the deprioritization of exonic disease-causing variants, especially missense variants or synonymous variants with a low-predicted impact on protein function but with potential to disrupt splicing. Well-defined clinical phenotypes also play an important role in the detection and better understanding of such variant types.

- Deep Exonic Variants

It has been estimated that the proportion of exonic splicing-relevant nucleotides may play a substantial role in disease [64]. Since the splicing effect of missense variants could be easily overlooked, well-defined clinical phenotypes combined with RNA-seq or minigene functional assays have a potential to unravel deep exonic missense variants located outside the exon-intron borders that impact splicing (Figure 1). For example, after negative predictions by several available machine learning-based computational tools used to explore the splicing effect of missense variants in patients with muscular dystrophy, limb-girdle, and autosomal recessive 1 (LGMDR1; OMIM: #253600), eight out of twenty-one deep exonic missense variants in *CAPN3* were shown to affect splicing using minigene assays [37] (Table 1). Similarly, to unravel the loss-of-function mechanism of missense variants in Gitelman syndrome (GTLMNS; OMIM: #263800), a minigene assay was used to show that several exonic missense variants in the *SLC12A3* affect mRNA splicing [38] (Table 1).

b. Synonymous Variants

Synonymous SNV (sSNV) refers to a coding region variant which results in no alternation of the amino acid sequence (Figure 1). Therefore, these variants are often disregarded as functionally irrelevant or “silent” genetic variations. However, these variants could affect splicing, transcription, and mRNA stability [65], yet the ability to predict the effects of sSNVs by computational methods is limited [66]. However, since aberrant splicing has been established as an important cause of RDs, research on the effect of sSNVs on splicing is growing. For example, in a patient with a clinical diagnosis of autosomal dominant seizures, benign neonatal, 1 (BFNS1; OMIM: #121200) but negative initial findings in the *KCNQ2*, ES re-analysis identified a rare synonymous variant in the corresponding gene *KCNQ2*. The effect on splicing was later confirmed using a minigene assay showing a prematurely truncated protein [39] (Table 1). The identification of a new synonymous variant in the *TAZ* gene in a Barth syndrome (BTHS; OMIM: #302060) patient, which alters *TAZ* mRNA splicing, also showed the functional importance of sSNVs [40] (Table 1). Furthermore, the above-discussed case of ataxia-telangiectasia is an important reminder that synonymous variants (maternally inherited *ATM* sSNV in that proband) may be combined with another complex variant, such as deep intronic variant (paternally inherited deep intronic in *ATM*), which is something to be considered in difficult-to-solve cases [21] (Table 1). 

#### 2.2.2. Complex Variants

- MEIs

In addition to MEIs in the intronic regions, MEIs in coding regions may be missed by standard clinical testing. For example, in a proband with a clinical diagnosis of autosomal recessive Alstrom syndrome (ALMS; OMIM: #203800) and only a single paternally inherited stop-gain variant, targeted LRS identified an Alu insertion in exon 20 of *ALMS1* as a second missing pathogenic variant [15] (Table 1). Similarly, in a patient with Bardet-Biedl syndrome 1 (BBS1; OMIM: #209900) who carried only one maternal missense allele in the *BBS1* gene, a novel ~1.7-kb retrotransposon insertion in exon 13 on paternal chromosome was identified using SR-GS [41] (Table 1).

b. GRs

GRs can affect protein-coding regions as well, and albeit CNVs are well-captured using arrays, SVs and CGRs could be missed for various reasons. For example, while no mutation could be identified in the *ALPL* gene in a patient with autosomal recessive hypophosphatasia (HPP) (HPPC; OMIM: #241510/HPPI; OMIM: #241500/HPPA; OMIM: #146300), quantitative polymerase chain reaction (qPCR) analysis found a novel homozygote duplication encompassing exons 2 to 6 of *ALPL* after negative results by ES [42] (Table 1). In two probands with unsolved autosomal dominant Wagner syndrome 1 (WGN1; OMIM: #143200), using targeted SR-GS, real-time qPCR, and long-range PCR, heterozygous deletions in exon 8 of *VCAN* were identified [43] (Table 1). After no candidate genes were identified by ES in a patient with lymphoproliferative syndrome, X-linked, 1 (XLP1; OMIM: #308240), an extended ES analysis of the *SH2D1A* identified breakpoints in exon 2 and intron 2 which were further validated to be a CGR including two deletions and one inversion [44] (Table 1). In a cohort of patients with a highly suggestive clinical diagnosis of autosomal recessive Johanson-Blizzard syndrome (JBS; OMIM: #243800), MLPA analysis of the *UBR1* was applied in patients with an unsolved genotype with negative results or only a single variant. Exon deletions/duplications were identified in these unsolved or partially solved patients, confirming the clinical diagnosis and increasing the molecular diagnostic rate [45] (Table 1).

c. Mosaic Variants

Genetic mosaicism results from the co-existence of genetically distinct cells in an individual due to postzygotic mutational events [67] (Figure 1). The established clinical phenotypes associated with mosaicism are especially important when considering that variants can arise in tissues other than blood. For example, disease-associated mosaic variants were identified at a frequency of ~1–1.5%, but when it comes to diseases such as epilepsy-related neurodevelopmental disorders, it could be as high as 3% of the pathogenic mosaic variants identified [68,69]. Especially in some cases, the somatic variant is a second variant and functions in a biallelic 2-hit mutational mechanism, giving rise to a clinical phenotype by a somatic and an original germline variant together [70]. 

Epilepsy-associated disorders offer a valuable opportunity to study the 2-hit mechanisms of a disease. The brain-tissue-specific mosaic variants during cortical development could give rise to brain malformations or non-lesional focal epilepsy [70]. So far, most of the research has been focused on second hit variants in coding regions. For example, a maternally inherited *DEPDC5* nonsense variant was detected in a blood sample by capture sequencing, while a second nonsense somatic variant was detected by conventional Sanger sequencing in the postoperative brain tissue of child with focal cortical dysplasia type II (FCORD2; OMIM: #607341) [46] (Table 1). Beyond epilepsies, mosaic variants contribute to other rare diseases. One example is a complex mosaic variant recently reported in a child with spastic paraplegia-4 disorder (SPG4; OMIM: #182601). The proband presented with spastic paraplegia along with autism and dysmorphisms, which had indicated a more complex genetic mechanism. ES revealed a combination of de novo mosaic bi-alternative variants in *SPAST* and a copy number variant [47] (Table 1). In patients with hyperinsulinemic hypoglycemia, familial, 1 (HHF1; OMIM: # 256450), a paternally inherited *ABCC8* nonsense variant was identified by Sanger sequencing without the identification of a second variant. A somatic maternal loss of heterozygosity at 11p15 was found by the further analysis of microsatellite markers in resected pancreatic tissue [48] (Table 1). However, while research efforts continue to report on protein-coding mosaic variants in various types of RDs such as epilepsy, cerebral cavernous malformation, focal cortical dysplasia type IIb/hemimegalencephaly, hypothalamic hamartoma, and others, mosaic variants in non-coding regions are still underreported—presumably due to technical and cost challenges associated with the depth of the SR-GS required and variant interpretation challenges [71].

d. Oligogenic Inheritance

Digenic inheritance is the major and simplest form of oligogenic inheritance, where two non-allelic mutations of functionally-related genes are co-inherited to elicit the clinical phenotype [72] (Figure 1). So far, 223 OMIM entries include the term “digenic” and 85 include “oligogenic” (accessing date: 2nd December 2022). 

Since the first report of digenic variants in retinitis pigmentosa 7 (RP7; OMIM: #608133) in 1994, where heterozygous mutations in two separate loci in ROM1 and RDS gene were described in RP7, the digenic inheritance has been one of the vital mechanisms in RDs [73]. The established DIgenic Diseases DAtabase (DIDA) via http://dida.ibsquare.be accessed on 1 December 2022 compiles detailed information on 258 reported digenic combinations corresponding to 54 different digenic diseases and has been described since 1994 [74,75]. In 26 unrelated families with genetically unsolved holoprosencephaly 1 (HPE1; OMIM: #236100) after conventional diagnostic procedures, 10 families were found to have oligogenic events with both known and novel holoprosencephaly genes by ES [49] (Table 1). After the identification of *PKP2* mutations by diagnostic tests in two arrhythmogenic cardiomyopathy (ACM), with the hypothesis of digenic inheritance, more candidate genes including *TTN* which co-segregated with *PKP2* variants were identified by further ES [50] (Table 1). The consideration of oligogenic mechanisms extends further than germline, with increasing evidence of 2-hit models and somatic variants playing a role in oligogenic tissue-specific disease mechanisms. For example, after a germline pathogenic truncation variant in *NPRL3* was identified in a patient with focal cortical dysplasia type II (FCORD2; OMIM: #607341), a somatic missense variant in the *WNT2* gene in brain-derived DNA was detected [51] (Table 1).

## 3. Functional Confirmation of the Role of Complex Genetic Mechanisms in RDs 

The increased use of different sequencing methods in clinical settings must be accompanied by increased access to confirmatory experiments. Functional confirmation could provide critical support to turn a possible diagnosis into a definite diagnosis [76]. There is heterogeneity and challenges to the accessibility of the collection of biomedically relevant and quality assessed clinical samples from RD patients, such as blood, plasma, DNA, RNA, and pathological tissue specimens. Therefore, fast and cost-effective processes are needed to confirm the complex rare genetic mechanisms in RDs and contribute to targeted treatment. Up to 50% of the mechanisms underlying human genetic disorders could be mutations involved in the splicing process [77,78]. Timely targeted transcript tests and reverse transcription PCR (RT-PCR) may be applied for aberrant RNA products [21] or transcriptome analyses may be applied when suspecting more complex transcript effects [27]. However, when the relevant tissue is not directly available from the patients, or the samples do not have enough quantity or quality, in vitro experiments including minigene assays or human induced pluripotent stem cells (iPSC) may be needed to confirm the functional effects.

For example, to prove the transcript effects of a large intronic transposal insertion in *SLC17A5,* RNA analysis was needed to show that this variant caused the inclusion of two aberrant splice sites, resulting in premature translation through frameshifts [11]. Intronic variants in *FBN1* were also proven to cause intron retention by RNA-Seq in Marfan syndrome (MFS; OMIM: #154700) family [79]. In clinically well-established syndromes such as HDBSCC and POLR3-HLD, deep intronic variants were confirmed to disrupt mRNA splicing by including a novel splice acceptor site, a pseudoexon, using targeted RNA-Seq, RT-PCR [80,81].

Further protein expression analysis or metabolomics study could also help support the pathogenicity of the variant by testing the functional impact on genes or gene products [76]. For example, the 5′-UTR variant of the *AR* gene in an AIS patient was found to alter gene function not by affecting RNA expression but by reducing AR protein levels [24]. The analyses of both RNA and protein expression in patients with Nonaka myopathy (NM; OMIM: #605820) showed a reduced expression of the affected allele caused by promoter region deletion [32].

In vitro model systems also play an important role in confirming a functional impact. Cell lineages and organoids differentiated from iPSCs could be powerful approaches to elucidate the pathologic impact of complex genetic mechanisms, especially where access to relevant tissues is difficult. The patients’ cell-derived organoid culture produces living mutant cells, providing possibilities for downstream validation experiments and multi-omics profiling [82]. For example, to investigate the mechanism of autosomal dominant retinitis pigmentosa (RP; OMIM: #268000), iPSCs were reprogrammed from patients’ fibroblasts and differentiated into retinal organoids. An altered TAD structure caused by SVs was identified using Hi-C combined with RNA-Seq [35]. 

## 4. Clinical Implications

By summarizing some of the examples here, we intended to highlight the power of clinical diagnosis in helping us decipher complex disease mechanisms in RDs and the implications for advanced methods to uncover genetic mechanisms in patients where genetic diagnosis is proving difficult. It is important that efforts continue to be invested in finding missing variants in often-overlooked RD cases with definite or highly suspected clinical diagnosis, where clinical genetic tests are unable to identify the genetic cause of the disease. Identifying the missing variants in these patients has the potential to help in decision making regarding the appropriate technology to be applied and the development of analytical methods. Currently, both LRS and SR-GS are reporting success in identifying missing variants in these patients, including MEIs, CNVs, SVs, CGRs, and SNVs (Table 1). It is clear that most of the missing variants reported cannot be identified using ES technology and that SR-GS is necessary; however, it would be important to probe the instances where SR-GS in patients with a clinical diagnosis is unable to identify the variant but alternate technologies are, such as LRS or OGM, in order to fully understand the limitations of SR-GS. So far, SR-GS has been applied in clinical settings with the potential to reduce the number of tests performed as a one-test-for-all prospect because of its genome-wise detection capability of a vast range of variant types [83]. Although SR-GS could simplify the number of steps of the routine clinical genetic diagnostics by the ability of variant detection, the potential utility of SR-GS also depends on further advances in the interpretation of the complexities of genetic variants before it can be fully appreciated. The studies focused on patients with well-defined clinical diagnoses and complex mechanisms of the disease have also the potential to drive a better understanding of the functional components in the human genome and therefore the development of analytical methods for the interpretation of the impact of complex variants (e.g., TAD domain disruptions, deep intronic variants). 

Importantly, the unravelling of the complex genetic mechanisms in patients with clinical diagnoses and functional characterization will not only help clarify the diagnosis, contribute to genetic counselling, and allow prenatal diagnosis, but also shape clinical management and improve prognostication [78]. Complex genetic mechanisms provide evidence for early clinical management, adjusting existing therapy strategies and adopting new therapeutics [84]. For example, in some RDs caused by an enzyme deficiency, early molecular diagnosis could benefit the rapid intervention with enzyme replacement treatment (ERT). When the causative homozygote duplication was identified by qPCR after the negative results of the Sanger sequencing, the life-saving ERT for a severe hypophosphatasia (HPP) (HPPC; OMIM: #241510/HPPI; OMIM: #241500/HPPA; OMIM: #146300) patient was started, and the symptoms such as respiratory failure improved during the treatment [42]. In MPS VI caused by a lysosomal enzyme deficiency, an early genetic diagnosis by clarifying the deep intronic causative variants could support early clinical interventions to guarantee treatment success and potential therapeutic strategies such as enzyme replacement therapy [85]. Genetic mechanisms are increasingly being leveraged to develop the personalized treatment of RDs by customizing sequence-specific drug targets. By unravelling the different complex genetic mechanisms of RDs, targeted treatments against the disease gene or encoded mRNA could be applied accordingly. The antisense oligonucleotide drug Milasen was developed to correct mis-splicing caused by an *MFSD8* SVA (SINE–VNTR–Alu) insertion in ceroid lipofuscinosis, neuronal, 7 (CLN7; OMIM: #610951) [27]. For the deep-intronic variant that created cryptic donor splice sites in *ATM*, in vitro experiments proved that by using an antisense morpholino oligonucleotide, the splice site was masked and the functional ATM kinase impairment was reverted [86]. For REs, the CRISPR/Cas9 system could be a promising way to repair the variants. For example, the *Streptococcus pyogenes* nuclease variant has been adapted to target the expanded CAG repeat tract in Huntington’s disease (HD; OMIM: #143100), which led to the rescue of the phenotypic abnormalities of differentiated neurons in HD-mouse embryonic stem cells [87]. Therefore, identifying complex genetic mechanisms offers opportunities to develop highly individualized and targeted treatments for specific variant types.

In summary, the clinical-diagnosis-driven strategy can significantly facilitate and guide molecular diagnostics, especially for elucidating complex genetic mechanisms that will further contribute to the development of confirmatory experiments and clinical management. Although different methods were applied to unravel complex genetic mechanisms, it is high time to have an optimized sequencing strategy that is simplified, comprehensive, and feasible for clinical routine use. With the help of known clinical diagnoses to elucidate complex genetic mechanisms, the application of genome-scale technologies and the further functional exploration by transcriptome sequencing and epigenomics could contribute to a better understanding of the nature of the genetic mechanisms in RDs, as well as the ways to detect and interpret them more efficiently [88].

## Figures and Tables

**Table 1:** The examples of clinical diagnoses leading to the elucidation of the complex genetic mechanism.

Author	Publication Year	Clinical Diagnosis	Inheritance	Causative Gene	Previous Genetic Tests	Previous Incomplete Genetic Findings	Further Analysis to Make a Definite Molecular Diagnosis	Further Genetic Findings	PMID
Wooderchak-Donahue, WL. et al. [20]	2018	hereditary hemorrhagic telangiectasia (HHT) (HHT1; OMIM: #187300/HHT2; OMIM: #600376/Juvenile polyposis/HTT syndrome; OMIM: #175050)	AD	*ENG, ACVRL1, SMAD4*	ES	negative results	SR-GS, SR-GS panel sequencing	deep intronic variants, one translocation in *ACVRL1*	30244195
Maroilley, T. et al. [21]	2022	ataxia–telangiectasia (AT; OMIM: #208900)	AR	*ATM*	clinical ataxia gene panel	Heterozygous synonymous variant	SR-GS	deep intronic variant	35145552
Whiffin, N. et al. [22]	2020	neurofibromatosis, type 2 (NF2; OMIM: #101000)	AD	*NF2*	targeted sequencing	negative results	analyzing targeted sequencing data	5′ untranslated region variants	32461616
Wright, C. F. et al. [23]	2021	neurodevelopmental disorder with hypotonia, stereotypic hand movements, and impaired language (NEDHSIL; OMIM: #613443)	AD	*MEF2C*	ES	negative results	ES data analysis	5′ UTR variants	34022131
Hornig, NC. et al. [24]	2016	androgen insensitivity syndrome (AIS; OMIM #300068)	XLR	*AR*	Sanger sequencing	negative results	SR-GS of AR genomic locus	5′UTR variant	27110943
Schnappauf, O. et al. [25]	2020	vasculitis, autoinflammation, immunodeficiency, and hematologic defects syndrome (VAIHS; OMIM: #615688)	AR	*ADA2*	ES/Sanger sequencing of *ADA2*, chromosomal microarray, ES	heterozygosity for the known pathogenic variant in one family/negative results in the other family	SR-GS/MLPA in combination with long-read PCR sequencing	5′UTR variant/a homozygous 800bp duplication	32638197
Bhatia, S. et al. [26]	2013	aniridia (AN1; OMIM: #106210)	AD	*PAX6*	ES, array-CGH, MLPA testing	negative results	screening a selection of eye-related cis-regulatory elements	cis-element (SIMO enhancer) variant	24290376
Tarailo-Graovac, M. et al. [11]	2017	sialic acid storage disease (SASDs) [infantile sialic acid storage disease (ISSD; OMIM: #269920)/Salla disease (SD; OMIM: #604369)]	AR	*SLC17A5*	Sanger sequencing	no pathogenic variant	ES, RNA and genomic DNA analysis	homozygous 6040 bp intronic transposal insertion in intron 9 of *SLC17A5*	28187749
Kim, J. et al. [27]	2019	ceroid lipofuscinosis, neuronal, 7 (CLN7; OMIM: #610951)	AR	*MFSD8*	genetic panel testing (including deletion–duplication analysis)for known Batten’s disease genes	single known missense variant	SR-GS	an insertion of an SVA (SINE–VNTR–Alu) retrotransposon	31597037
van Kuilenburg, ABP. et al. [7]	2019	global developmental delay, progressive ataxia, and elevated glutamine (GDPAG; OMIM #618412)	AR	*GLS*	ES	missense in patient 1 and a duplication variant in patient 3	SR-GS	GCA trinucleotide expansion	30970188
LaCroix, A. J. et al. [28]	2019	Baratela-Scott syndrome (BSS; OMIM: #615777)	AR	*XYLT1*	ES, clinical chromosome microarray, Sanger sequencing	single or no causative variants in *XYLT1*	Southern Blot and SR-GS analysis	GGC repeat expansion	30554721
Ishiura, H. et al. [8]	2018	epilepsy, familial adult myoclonic (FAME)	AD	*SAMD12, TNRC6A, RAPGEF2*	analysis of the exons of 38 genes located in the candidate region, including copy-number analysis	negative results	single-molecule, real-time sequencing of BAC clones and nanopore sequencing	expansions of TTTCA and TTTTA repeats	29507423
Corbett, M. A. et al. [29]	2019	epilepsy, familial adult myoclonic, 2 (FAME2; OMIM: #607876)	AD	*STARD7*	NA	NA	SR-GS	ATTTC repeat expansions	31664034
Florian, R. T. et al. [30]	2019	epilepsy, familial adult myoclonic, 3 (FAME3; OMIM: #613608)	AD	*MARCH6*	ES	negative results	SR-GS and repeat-primed PCR	intronic TTTTA/TTTCA expansions	31664039
Yeetong, P. et al. [31]	2019	benign adult familial myoclonic epilepsy type 4 (FAME4; OMIM: #615127)	AD	*YEATS2*	targeted resequencing of the 10-Mbp critical region, array CGH, ES and SR-GS	negative results	single-molecule real-time sequencing	TTTCA repeat insertions	31539032
van Kuilenburg, ABP. et al. [13]	2018	dihydropyrimidine dehydrogenase deficiency (DPD deficiency; OMIM: #274270)	AR	*DPYD*	Sanger sequencing	heterozygous missense variant	SR-GS	large intragenic inversion	29691939
Garland, J. et al. [32]	2017	Nonaka myopathy (NM; OMIM: #605820)	AR	*GNE*	Sanger sequencing	heterozygous mutation	copy number variant analysis of *GNE*	deletion in the promoter region	28717665
Schanze D, et al. [33]	2014	Marshall-Smith syndrome (MRSHSS; OMIM: #602535)	AD	*NFIX*	conventional sequencing of *NFIX*	causes for part of the patients including frameshift and splice-site mutations	MLPA	a recurrent large deletion	24924640
Todorov T, et al. [34]	2016	Wilson disease (WD; OMIM: #277900)	AR	*ATP7B*	extensive sequence analysis of promoter, coding region and associated intron-exon boundaries	negative results	selective amplification and MLPA	intragenic deletions	27992490
de Bruijn, S. E. et al. [35]	2020	retinitis pigmentosa (RP; OMIM: #268000)	AD	genomic region spanning *YPEL2* to LINC01476	SR-GS	SVs	Hi-C	topological-associated domains	33022222
Gueant, JL. et al. [36]	2018	methylmalonic aciduria and homocystinuria, cobalamin C type (MAHCC; OMIM: #277400)	AR	*MMACHC*	Sanger sequencing	single heterozygous mutations	methylation analysis (Sanger sequencing of bisulfite-converted DNA)	heterozygous promoter hypermethylation	29302025
Dionnet, E. et al. [37]	2020	muscular dystrophy, limb-girdle, autosomal recessive 1 (LGMDR1; OMIM: #253600)	AR	*CAPN3*	present machine learning-based computational tools	negative predictions	minigene assay	deep exonic missense variants	32668095
Takeuchi, Y. et al. [38]	2015	Gitelman syndrome (GTLMNS; OMIM: #263800)	AR	*SLC12A3*	previously reported or are accessible from the PubMed database	missense variants	minigene assay	exonic variants affecting mRNA splicing	25060058
Li, Q. et al. [39]	2021	seizures, benign neonatal, 1 (BFNS1; OMIM: #121200)	AD	*KCNQ2*	ES	negative results	ES reanalysis	synonymous variant	34107977
Ferri, L. et al. [40]	2016	Barth syndrome (BTHS; OMIM: #302060)	XLR	*TAZ*	NA	NA	sequencing of the *TAZ* gene	new synonymous variant	26853223
Miller, DE. et al. [15]	2021	strongly suspected clinical diagnoses such as Hermansky-Pudlaksyndrome (HPS1; OMIM: # 203300), glycogen storagedisease III (GSD3; OMIM: #232400) etc.	AR, X-linked	*ALMS1,NPHP4,VARS2 etc.*	chromosomal microarray, karyotype, clinical ES, or research SR-GS	single variant missed in a recessive condition or no variants found in an X-linked condition	T-LRS	deletions, mobile element insertions, inversions, repeat expansions, and intronic variants predicted to affect splicing	34216551
Tavares, E. et al. [41]	2019	Bardet-Biedl syndrome 1 (BBS1; OMIM: #209900)	AR	*BBS1*	SR-GS on 19 BBS genes	missense allele	SR-GS	Novel ~1.7-kb retrotransposon insertion	30484961
Hacıhamdioğlu, B. et al. [42]	2019	Hypophosphatasia (HPP) (HPPC; OMIM: #241510/HPPI; OMIM: #241500/HPPA; OMIM: #146300)	AR	*ALPL*	ES	negative results	quantitative PCR	large duplication	30468149
Burin-des-Roziers, C. et al. [43]	2016	Wagner syndrome 1 (WGN1; OMIM: #143200)	AD	*VCAN*	Sanger sequencing	no nucleotide variations at exon 8 boundaries	targeted deep SR-GS, quantitative real-time PCR, and long-range PCR	heterozygous deletions	27667122
Wu, L.et al. [44]	2022	lymphoproliferative syndrome, X-linked, 1 (XLP1; OMIM: #308240)	XLR	*SH2D1A*	ES	negative results	extended ES analysis	complex structural variant including two deletions and one inversion	35092357
Sukalo M, et al. [45]	2017	Johanson-Blizzard syndrome (JBS; OMIM: #243800)	AR	*UBR1*	Sanger sequencing	negative results or only a single variant	MLPA	exon deletions/duplications	29178640
Ribierre, T. et al. [46]	2018	focal cortical dysplasia type II (FCORD2; OMIM: #607341)	2-hit genetic model	*DEPDC5*	deep sequencing of a panel of mTORC1 genes	heterozygous variant in blood	Sanger sequencing	brain somatic variant	29708508
Matthews, A. M. et al. [47]	2017	spastic paraplegia-4 disorder (SPG4; OMIM: #182601)	AD	*SPAST*	chromosome microarray	copy number variant	ES, pyrosequencing	de novo mosaic bi-alternative variants	28778789
Joyce, C. M. et al. [48]	2020	hyperinsulinemic hypoglycemia, familial, 1 (HHF1; OMIM: # 256450)	2-hit genetic model	*ABCC8*	Sanger sequencing	paternally inherited *ABCC8* nonsense variant	further analysis for microsatellite markers	somatic maternal loss of heterozygosity at 11p15	32695361
Kim, A. et al. [49]	2019	holoprosencephaly 1 (HPE1; OMIM: #236100)	AD	180 genes directly linked to the SHH signalling, cilium and Wnt/PCP pathways	targeted HPE gene-panel sequencing, CGH, MLPA	negative results	ES	oligogenic variants	30508070
Konig, E. et al. [50]	2017	arrhythmogenic cardiomyopathy (ACM)	digenic inheritance	*PKP2* and *TTN*	diagnostic tests	*PKP2* mutations	ES	*TTN* mutations	29221435
Bennett, MF. et al. [51]	2022	focal cortical dysplasia type II (FCORD2; OMIM: #607341)	2-hit genetic model	mTOR and related pathway genes	ES	truncating variant in *NPRL3*	ES	mosaic missense variant in brain-derived DNA in the *WNT2* gene	35097204

Abbreviations: CGH, comparative genomic hybridization; ES, exome sequencing; Hi-C, high throughput chromosome conformation capture; LRS, long read sequencing; MLPA, multiplex ligation-dependent probe amplification; mTOR, mammalian target of rapamycin; NR, not reported; PCR, polymerase chain reaction; SR-GS, short-read genome sequencing; T-LRS, targeted long read sequencing.

## Data Availability

Not applicable.

## Abbreviations

AIS: androgen insensitivity syndrome; ALMS: Alstrom syndrome; AN1: aniridia; AT: ataxia–telangiectasia; BFNS1: seizures, benign neonatal, 1; BSS: Baratela-Scott syndrome; BTHS: Barth syndrome; CGRs: complex genomic rearrangements; CLN7: ceroid lipofuscinosis, neuronal, 7; CNVs: copy number variants; DPD: dihydropyrimidine dehydrogenase; ERT: enzyme replacement treatment; ES: exome sequencing; FAME: epilepsy, familial adult myoclonic; FCORD2: focal cortical dysplasia type II; SPG4: spastic paraplegia-4 disorder; GDPAG: global developmental delay, progressive ataxia, and elevated glutamine; GRs: genomic rearrangements; GTLMNS: Gitelman syndrome; HHF1: hyperinsulinemic hypoglycemia, familial, 1; HPE1: holoprosencephaly 1; HPP: hypophosphatasia; HTT: hereditary hemorrhagic telangiectasia; iPSC: induced pluripotent stem cells; ISSD: infantile sialic acid storage disease; JBS: Johanson-Blizzard syndrome; LGMDR1: muscular dystrophy, limb-girdle, autosomal recessive 1; LRS: long-read sequencing; MAHCC: methylmalonic aciduria and homocystinuria, cobalamin C type; MEIs: mobile element insertions; NEDHSIL: neurodevelopmental disorder with hypotonia, stereotypic hand movements, and impaired language; MFS: Marfan syndrome; MLPA: multiplex ligation-dependent probe amplification; MSS: Marshall-Smith syndrome; NF2: neurofibromatosis, type 2; NM: Nonaka myopathy; OGM: optical genome mapping; qPCR: quantitative polymerase chain reaction; RDs: Rare diseases; REs: repeat expansions; RP: retinitis pigmentosa; RT-PCR: reverse transcription PCR; SNVs: single nucleotide variants; SASDs: sialic acid storage disease; SD: Salla disease; SR-GS: short-read genome sequencing; sSNV: Synonymous SNV; SVs: structural variants; TADs: topologically associating domains; UTRs: untranslated regions; VAIHS: vasculitis, autoinflammation, immunodeficiency, and hematologic defects syndrome; WD: Wilson disease; WGN1: Wagner syndrome 1; XLP1: lymphoproliferative syndrome, X-linked, 1.
